# Adaptation of the Copenhagen Burnout Inventory in Latvia: Psychometric Data and Factor Analysis

**DOI:** 10.3390/ijerph22050761

**Published:** 2025-05-12

**Authors:** Olga Cerela-Boltunova, Inga Millere, Ingrida Trups-Kalne

**Affiliations:** 1 Department of Nursing and Midwifery, Riga Stradiņš University, LV-1067 Riga, Latvia; 2 Psychology Laboratory, Institute of Public Health, Riga Stradiņš University, LV-1067 Riga, Latvia

**Keywords:** burnout, Latvia, factor analysis, healthcare, face validity

## Abstract

Burnout is a widespread occupational phenomenon with adverse effects on the well-being and performance of healthcare professionals. In Latvia, the lack of a psychometrically validated instrument for measuring burnout has hindered effective assessment and intervention. This study aimed to adapt the Copenhagen Burnout Inventory (CBI) for use in the Latvian context and to evaluate its psychometric properties among healthcare workers. A cross-sectional study was conducted in Latvia with a total of 288 participants from various healthcare institutions. The adaptation process included forward translation, expert panel review, and face validity testing. The initial item pool comprised 19 items reflecting three subscales: personal burnout (PB), work-related burnout (WB), and client-related burnout (CB). Reliability was assessed using Cronbach’s alpha, composite reliability (CR), and average variance extracted (AVE). The final model showed strong internal consistency (α > 0.80), acceptable construct validity (CR > 0.80; AVE > 0.50), and a good model fit (χ^2^/df = 2.6; RMSEA = 0.06; CFI = 0.95; TLI =0.94). The findings demonstrate that the Latvian version of the CBI is a valid and reliable tool for assessing burnout among healthcare professionals. This study represents the first full adaptation and validation of the CBI in Latvia and provides a foundation for future research and practical applications in occupational health monitoring and burnout prevention.

## 1. Introduction

Burnout, also known as emotional exhaustion syndrome [1], has become one of today’s major psychological and social challenges, especially in the work environment [2]. It is characterised by physical and emotional exhaustion resulting from prolonged and intense stress associated with high workloads, demanding responsibilities, and inadequate support [3,4]. The International Classification of Diseases, ICD-11, defines burnout as a syndrome resulting from chronic work stress that has not been successfully managed [5]. The syndrome has three main dimensions: emotional exhaustion, depersonalisation, and reduced work performance [3,4,6].

Today, burnout is not only an individual problem but also a socio-economic phenomenon [2], affecting the productivity of organisations and public health in general [7]. Studies [8,9,10] show that burnout is particularly common in caring professions such as healthcare [8], education [9], and social work [10]. In these sectors, workers are constantly in close contact with people, which increases emotional strain and stress [8,11]. Therefore, these sectors are becoming the main targets for burnout research and prevention [12].

In Latvia, burnout has become a particularly important issue in recent years, especially in the healthcare sector [13]. The healthcare system is faced with understaffing, continuous reforms, and high demands on healthcare workers [14]. During the pandemic, these problems were exacerbated, leading to increased stress, emotional exhaustion, and an inability to maintain a work–life balance [15]. Many healthcare workers in Latvia face an excessive workload [16]. Many healthcare professionals work more than one full-time job to compensate for low salaries and staff shortages [17]. They receive insufficient support from management and organisations [18]. Workers often experience insufficient emotional and practical support, and chaos and uncertainty in the work process increase stress and negative attitudes to work [15,16].

According to several studies, burnout is very common among Latvian healthcare workers [19,20,21]. For example, a study conducted in 2020 [21] found that almost 50% of nurses in Latvia experience high levels of emotional burnout, while almost a third experience symptoms of depersonalisation. Similar tendencies are observed in other professions that require intense emotional involvement [20]. One of the main problems in Latvia is insufficient understanding of the nature of burnout and a lack of effective solutions [22]. The diagnosis of burnout is often neglected as it is considered by many to be a normal part of the work process [20]. As a result, there is a lack of a systematic approach to both the prevention and elimination of burnout [23].

Although the Maslach Burnout Inventory (MBI) is considered one of the most popular tools for assessing burnout [24,25], its use has several limitations, especially in the Latvian context. First, the MBI is a paid service that requires significant financial resources from both researchers and organisations [26]. In Latvia, where research and organisational budgets are often limited, this factor significantly limits its availability [14]. This contributes to the need to find alternatives that are free but at the same time reliable and effective. Second, the MBI focuses primarily on three dimensions: emotional exhaustion, depersonalisation, and reduced professional efficacy [3]. Although these dimensions are important, they do not necessarily provide a complete picture of the problem of burnout in different cultural and professional settings [2]. For example, the MBI tends to be too specific to caring professions and its use in other sectors may be less effective [2]. Third, there are concerns about the suitability of the MBI for different cultures and languages [27]. Translations and adaptations often fail to reflect specific contexts and cultural nuances, which may affect the reliability and interpretation of results [28]. Therefore, there is a critical need to establish a culturally adapted, psychometrically sound version of the CBI for Latvian-speaking populations.

To address these issues, an alternative tool, the Copenhagen Burnout Inventory (CBI), was adopted in Latvia during this study. The CBI has several advantages: it is freely available to researchers and organisations, which allows it to be used more widely. It is suitable for different professional groups and contexts [29]. The CBI assesses personal burnout, work-related burnout, and client-related burnout. This approach allows a more accurate analysis of the causes of burnout and the development of targeted intervention strategies [30]. When adapting the CBI to the Latvian context, linguistic, and cultural specificities were taken into account, which improves the reliability and validity of the tool.

The Latvian research team decided to adapt the CBI to ensure effective diagnosis and assessment of burnout. This process included one-way translation, expert judgement, face validity testing, and psychometric analysis. The adaptation of the CBI may help to better understand the extent of the burnout problem [31] in Latvia and provide practical recommendations to alleviate it. Studies [32,33] conducted using the CBI show that the level of burnout among Latvian healthcare workers is comparable to that in other countries but emphasise the need for improved work organisation and support systems [16,17].

Burnout is a global problem that requires special attention [5], also in Latvia. Given the specificities of the Latvian healthcare system and other sectors, the choice of the Copenhagen Burnout Inventory is a strategically important step. Its accessibility, adaptability, and accuracy make it a valuable alternative to the Maslach questionnaire. The CBI provides a tool not only for diagnosing burnout but also for designing effective strategies to prevent it and improve the well-being of employees [34].

Latvia’s example can serve as good practice for other countries facing similar challenges and looking for affordable and reliable tools to tackle burnout. Further research and practical measures will help to improve the working environment and reduce the impact of burnout, contributing to both employee well-being and organisational productivity [35].

The purpose of this study is to adapt and validate the CBI in the Latvian context by conducting a thorough translation process, including expert evaluation and pilot testing, and by evaluating the psychometric properties of the instrument using a large sample of healthcare professionals. This includes analyses of internal consistency, content validity, and construct validity through CFA, as well as AVE and CR indices.

This study contributes to the literature by being the first to offer a systematically validated Latvian version of the CBI. The findings are expected to support the use of the CBI in research and practice in Latvia, enabling more accurate assessment of burnout levels and facilitating international comparisons.

## 2. Materials and Methods

### 2.1. Description of the Tool

The CBI is a standardised tool designed to measure burnout in different domains of life and work [33,36]. This tool focuses on the physical and psychological burnout of individuals, which is manifested in three main dimensions: personal burnout, work-related burnout, and client-related burnout [30].

The questionnaire consists of 19 items divided into three scales [30]. The first scale—personal burnout. This scale measures an individual’s general level of physical and psychological exhaustion, independent of specific external factors [37]. The second—work-related burnout, which measures exhaustion specifically attributed to the professional work environment [33]. And the third—client-related burnout, which assesses exhaustion resulting from direct interactions with clients, patients, or service users [30,37].

Only one or two scales may be used in a given study. This depends on the purpose of the study and the population. If part of the study population is not employed, only the personal burnout scale can be used. If part of the study population does not work with clients, two scales can be used, one for personal burnout and one for work-related burnout [30]. Some researchers [29,33,38] have compared the results of the three scales, but the authors of the survey [30] mention that this is not useful. Some researchers have combined the three CBI scales into an overall burnout scale [29,33,38], but this is a misconception.

Each item is scored on a five-point Likert scale [30]: Always (100); Often (75); Sometimes (50); Seldom (25); Never/almost never (0). In the original version, some items can be answered using a different scale: To a very high degree (100); To a high degree (75); To some extent (50); To a low degree (25); To a very low degree (0). For example, for questions such as: Do you feel tired at the end of the working day?

When adapting the tool to Latvian, we chose to use a single Likert scale with five levels for all items (Always, Often, Sometimes, Seldom, Never/Almost Never), a practical and logical solution that simplifies both the answering process and the analysis of the results. This unified framework can be easily adapted to already established methods and analysis tools, such as the calculation of means or the interpretation of dimension scores [39].

The scoring of the questionnaire is based on the individual analysis of each scale by calculating a mean score. These scores indicate the individual’s level of burnout in each dimension. Items with reversed scoring (e.g., item 10 ‘Do you have enough energy for family and friends during leisure time?’) require score inversion during analysis to maintain interpretive consistency [30].

According to the authors of the survey [30], it makes little sense to use a cut-off point to distinguish between those with burnout and those without. A cut-off point always leads to a loss of information, which is not desirable in research [40]. In some cases, and studies [41,42,43], however, colleagues have found it necessary to use a cut-off point to make the results easier to understand for non-target groups. For example, in Denmark and other countries [30,44,45], a cut-off point of 50 points has been used to identify the ‘high burnout’ group. However, there are no universal normative values based on representative samples across countries or populations. In Denmark, the average personal burnout score for working adults is around 32. For adults outside the labour market, the average score is around 37 [30]. In the PUMA study of workers doing ‘people work’ in hospitals, home care, and prisons, the average personal burnout score was 36, work-related burnout 33, and client-related burnout 31 [30].

The authors [30] mention that it is a misconception to ‘test’ items with factor analysis or similar statistical methods. The three scales are designed to be used in different contexts and to measure different concepts, not to create three independent scales in a given population [30]. However, there are several adaptation and validation studies [29,33,38] that have tested factor analysis and found that the scales are correlated and dependent. In the present study, we will also use factor analysis to test for scale dependence.

### 2.2. Method

The aim of this study was to adapt the Copenhagen Burnout Inventory (CBI) to the Latvian healthcare context, ensuring its relevance and psychometric soundness for use among Latvian health professionals. A cross-sectional design was applied to collect quantitative data, and the adaptation process followed internationally acknowledged methodological principles for instrument translation and validation, while also making contextually justified methodological decisions.

The study was conducted in two main phases: an instrument adaptation phase (including translation, expert validation, and face validity assessment) and a psychometric analysis phase (including reliability testing and construct validity analysis using pilot data from Latvian healthcare professionals). The study involved healthcare institutions across Latvia, with participants recruited through institutional cooperation.

The CBI is a free-to-use tool developed in Denmark and available in English. For this study, we performed a single forward translation from English to Latvian, conducted independently by three Latvian healthcare professionals fluent in both languages and experienced in clinical terminology. The choice of a single direction translation approach rather than the full translation/back-translation process, was guided by the practical objective of ensuring clarity, contextual relevance, and terminological precision in the target language. Another independent translator, unfamiliar with the original tool, then translated the Latvian version back into English to check that the original meaning was retained.

This decision is supported by several researchers who argue that back-translation does not always guarantee improved conceptual equivalence and may even introduce inconsistencies due to ww-reliance on literal equivalence rather than functional and con-textual fit.

Following the initial translation, the Latvian version of the CBI was reviewed by three independent experts in healthcare and psychology. These experts assessed each item for content validity, relevance, clarity, and comprehensibility. The Content Validity Index (CVI) was used to quantify agreement across experts. This process ensured that items were not only linguistically accurate but also culturally appropriate and understandable for the target population.

The choice of expert review over cognitive interviewing in this phase was based on practical and scientific reasons. The expert panel included practitioners with extensive experience in survey-based assessment and burnout research, making them well suited to judge item interpretability and clarity from both respondent and theoretical perspectives. This approach allowed a more efficient and focused evaluation of item quality in the context of healthcare professionals, where time and access are limited. Each expert was asked to rate the relevance of each item to the concept of burnout, using a scale where 0 meant ‘not applicable’ and 1 meant ‘applicable’. After receiving the data from the experts, the overall CVI was found to be 0.803. At the same time, items with a value below 0.6 were identified. According to Lynn’s guidelines [46], items with a CVI below 0.6 are considered to be incorrect and need to be revised or modified.

Items 1, 5, 8, 11–14, and 18 were revised and linguistically adapted without changing the essence of the items. As a result, a second version of the questionnaire in Latvian was obtained and the second phase of the CVI was launched. Some items were rephrased to make them easier to understand and to more clearly represent the concept of burnout. Additionally, more precise Latvian language terms were used to ensure consistency with both the original text and Latvian linguistic norms, and some items were rearranged to avoid potential meaning duplication across different scales. The same three experts who participated in the first phase were invited again for this phase. They were again asked to assess the relevance of the items to the concept of burnout. After data analysis, it was concluded that the CVI value had increased to 0.896 and there were no more items with a value below 0.6. The second version of the questionnaire in Latvian was not changed.

To further test the clarity and comprehensibility of the translated questionnaire, a face validity test was conducted among a small sample of healthcare professionals (n = 5) working in diverse care settings. Participants were asked to complete the questionnaire and provide feedback on the clarity and relevance of each item, as well as any difficulties in understanding or interpreting the content. Their responses confirmed that the translated items were understandable and perceived as relevant, supporting the appropriateness of the adapted instrument.

Face validity testing was considered a feasible and contextually appropriate alternative to full-scale cognitive interviews, which often require significant resources and time. In the Latvian context, where access to a large number of professionals for in-depth interviews is limited, this method provided a pragmatic and sufficiently rigorous approach to verifying respondent interpretation of the items.

After all these steps, we received the final version of the questionnaire in Latvian and the last step—factor analysis—was started. The respondents for the factor analysis were selected from hospitals and outpatient facilities all over Latvia. The selection criteria required participants to be at least 18 years old, have work experience in the healthcare sector (at least three months), and be certified in their specialty. The questionnaire was posted on the websites of various professional associations for easy access by respondents and via a secure online platform to ensure data confidentiality. The survey consisted of 12 demographic questions (age, gender, place of work, etc.) and 19 CBI questions. A total of 288 questionnaires were fully completed in the study, providing sufficient data for psychometric analysis.

**Figure 1:**
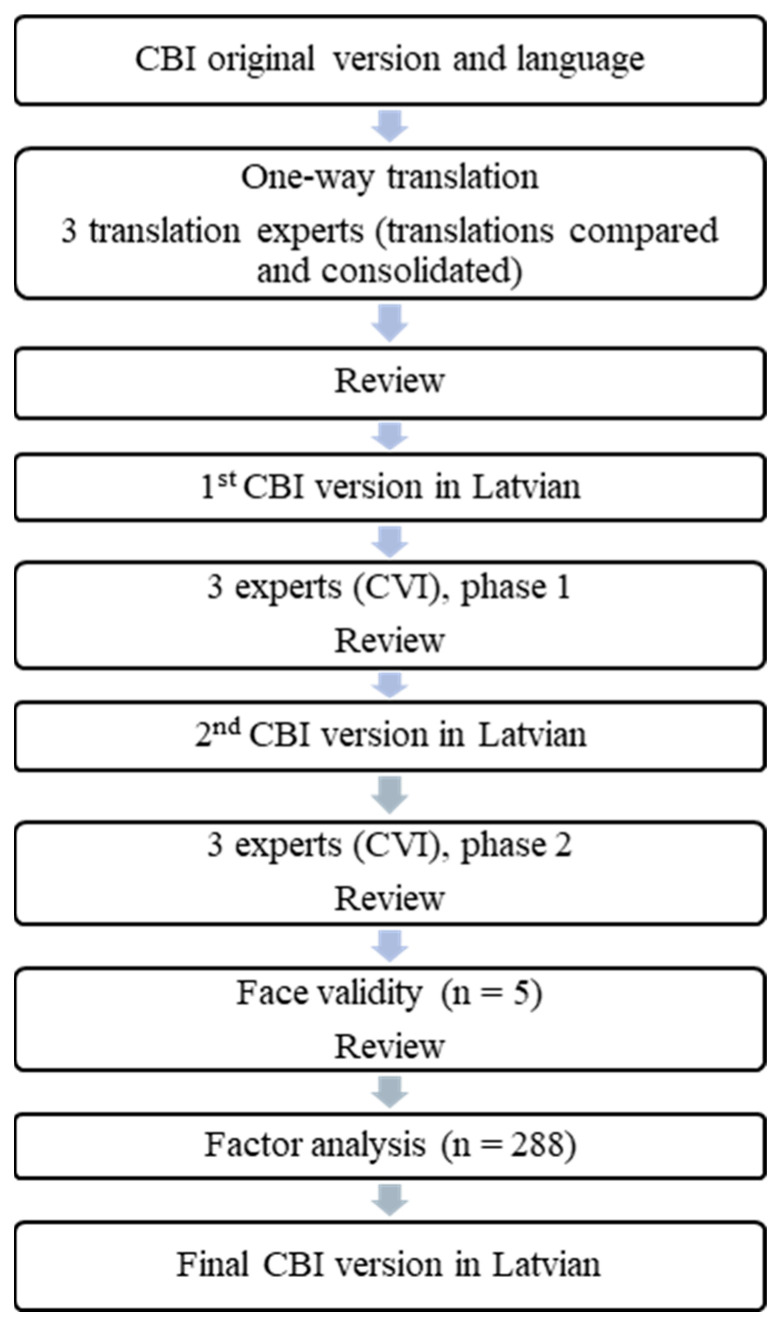
Adaptation process of Copenhagen Burnout Inventory.

Figure 1 illustrates the systematic and reflective approach to the adaptation and validation of the tool. Such a process is essential to ensure that the survey is valid and reliable in a new cultural and linguistic context [47,48].

### 2.3. Data Analysis and Statistics

Data analysis was carried out using IBM SPSS (version 28.0, IBM Corp., Armonk, NY, USA) and AMOS (version 24.0, IBM Corp., Armonk, NY, USA) to ensure a reliable and detailed assessment of the psychometric properties of the tool. The analysis included descriptive statistics, reliability tests, and factor analysis.

Means (M) and standard deviations (SD) were calculated for each of the three CBI scales and for individual items to provide an overview of participant demographics and burnout levels. Correlation coefficients (Pearson-r) were used to assess the relationship between dimensions and individual items. Demographic data were presented in tables including gender, age, occupational status, and working conditions to characterise the sample and analyse possible group differences.

Cronbach’s alpha was used to assess the internal consistency of each CBI dimension. McDonald’s omega was calculated as an additional reliability measure to test the robustness of the results under different conditions. Factor analysis was carried out in two steps: exploratory factor analysis (EFA—Kaiser–Meyer–Olkin) and confirmatory factor analysis (CFA—model goodness-of-fit indices—CFI, TLI, RMSEA, and correlations between dimensions plotted on a path diagram).

The results are presented in tables and graphs to clearly show the distribution of the data and the reliability of the tool. Tables include means and standard deviations, factor loads and uniqueness scores, and correlation tables between dimensions.

### 2.4. Participants

The study sample consisted of 288 healthcare professionals currently working in various medical institutions across Latvia. Participants were selected using non-probability purposive sampling to ensure representation of professionals directly involved in patient care. Inclusion criteria included: being at least 18 years old, having a minimum of three months’ professional experience in the healthcare sector, and being proficient in Latvian.

Most participants were recruited through professional organizations and institutional networks, with the survey distributed via secure online platforms. All participants voluntarily completed the questionnaire and provided informed consent.

The demographic characteristics of the sample—including gender, age, region, professional background, work environment, total workload, and work pattern—are described in detail in the Results section (see Table 3). The sample included a wide range of roles, such as nurses, physicians, physician assistants, midwives, and other allied healthcare professionals, providing a diverse cross-section of Latvia’s healthcare workforce. Although the majority of respondents were nurses and female professionals, this reflects the gender and professional distribution typical in Latvian healthcare.

### 2.5. Ethical Considerations

The study was conducted in accordance with the Declaration of Helsinki [49], which guarantees the protection of human rights of participants during the research process. Latvian national ethical requirements and laws and regulations on the collection and use of data in research were also observed.

The study was approved by the Ethics Committee of Rīga Stradiņš University (protocol code: 2-PĒK-4/416/2023, 9 May 2023). The Committee assessed the study methodology, participant recruitment process, and planned activities to ensure that the study complied with ethical standards.

## 3. Results

Three experts participated in the first and the second phase of adaptation, the average age of experts was 46.3 years. All the experts were women, two of whom had more than 10 years of experience. The experts had professional experience in direct patient care, research, knowledge transfer to students, and management.

The process of adapting the Copenhagen Burnout Inventory involved two stages of expert review to ensure content validity. Table 1 summarises the results of the expert review. Stage 1—CVI scores ranged from 0.33 to 1.00, with an overall CVI of 0.807. Individual items were revised, particularly those with a CVI of less than 0.67. Phase 2—after the revision, the CVI values improved significantly, with an overall CVI of 0.896. These results confirm the effectiveness of the revision.

After determining the CVI, the face validity (FVI) was determined for five respondents. The FVI was found to be 1, indicating high validity and reliability [47]. The mean age of the five respondents was 36.7 years. All professionals were female—two nurses, two physicians, and one physician assistant. They all came from different departments, profiles, and regions of the country; four of them had more than 10 years of professional experience. The experts have professional experience in direct care work with patients.

After completing all the stages, the final version of the scale in Latvian was obtained. The adapted scale can be found in Table 2.

In total, 288 respondents participated in the final phase of the study. The mean age was 42.13 years (SD = 12.18), with a range from 22 to 67 years. A total of 97.8% of the respondents were female, 1.75% were male, and 1% chose not to disclose their gender. The majority came from the Riga region (57.15%), followed by Vidzeme (17.05%). In total, 51.65% had a bachelor’s degree or equivalent, while 13.55% had a master’s degree. The majority of participants were nurses (81.3%), followed by medical assistants (7.35%) and other healthcare professionals. Years of experience ranged from 1 to 47 years, with a mean of 17.3 years (SD 11.74). A total of 59.35% worked in inpatient care, 30.95% in outpatient care, and the remainder in mixed or specialised settings. The demographic breakdown of the respondents is shown in Table 3.

Reliability analysis showed high internal consistency for all scales. McDonald’s omega was 0.948, indicating excellent reliability. The overall Cronbach’s alpha was 0.938. Each subscale showed strong consistency, demonstrating the reliability of the tool. The Cronbach’s alpha for the first scale was 0.920, the Cronbach’s alpha for the second scale was 0.791, and the Cronbach’s alpha for the third scale was 0.884.

The mean score of the personal burnout scale was 58.9 (SD = 17.3). This indicates a moderate level of burnout. All items (1–6) show a good to very good adjusted correlation with the total scale (0.67 to 0.85), except item 6, which has the lowest value (0.67). The Cronbach’s alpha value when removing the item increases only for item 6 (0.922), indicating that this item may be less consistent with the rest of the scale.

The mean score of the work-related burnout scale was 56.0 (SD = 15.8). Item 10 is problematic as it has a negative correlation with the total scale (−0.489). This may indicate that this item is not appropriate or not interpreted consistently with the others. With the exception of item 10, the remaining items show good adjusted correlations (0.628–0.785). The Cronbach’s alpha value increases significantly (0.914) when item 10 (‘Do you have enough energy for family and friends during leisure time?’) is removed, indicating the potential of this item to improve the scale. Importantly, however, it is item 10 that has the inverse significance mentioned by the authors of the scale [30].

The mean score for the client-related burnout scale was 48.9 (SD = 16.7). All items (14–19) show moderate to good adjusted correlations with the total scale (0.525–0.815). Item 17 has the lowest adjusted correlation (0.525) and Cronbach’s alpha increases to 0.898 when it is removed. This suggests that this item may be less relevant to the scale. The mean score for all three scales is 54.7 (SD 17.3). All the above results are shown in Table 4.

Table 5 shows the results of the exploratory factor analysis (EFA), which includes the three factors and the uniqueness scores. Each item (1–19) is related to one or more factors (1, 2, or 3). The loads indicate the extent to which each item is related to the factor. Values closer to 1 or −1 indicate a strong association, while values closer to 0 indicate a weak association. Uniqueness indicates the proportion of variation within each item that cannot be explained by the factors. Low uniqueness (closer to 0) indicates that the factors explain the item well. High uniqueness (above 0.5) may indicate that the item is poorly represented by the factors.

Some items (e.g., 4, 6, 13) show significant load on more than one factor, indicating conceptual overlap. Items 9 (0.261), 6 (0.516), and 18 (0.682) may be problematic as they are poorly represented by the factors. The negative load of item 10 indicates that it works against the direction of factor 1, which may require further investigation or explanation. Factors 1 and 2 are clearly defined, but factor 3 is less well expressed. The variance explained was 68% of the total variance. All items showed significant loads (>0.6) on their respective factors, with the exception of item 10. The Kaiser–Meyer–Olkin (KMO) goodness of fit for the whole sample was 0.942, indicating a good fit for the factor analysis. Further consideration should be given to rotation (e.g., varimax) or other statistical techniques to improve the interpretation of the factors.

Table 6 shows the results of the EFA based on eigenvalues. In this analysis, it was not specified exactly how many factors are in the scale. It was therefore suggested that the SPSS software should select and calculate the number of factors corresponding to this scale. This approach allows the software to automatically identify the most important factors that may have a significant impact on the results and conclusions of the analysis. In this calculation, a Factor 1 component and a Factor 2 component were defined.

Table 7 shows how the original components are rotated using the varimax rotation method with Kaiser normalisation. This table helps to interpret the factor structure after rotation, allowing a clearer understanding of the impact of the factors on the original data. Each entry shows the weight of the original component in the new (rotated) coordinate system. The coefficients describe how the rotation transforms the original component structure. Varimax rotation maximises the variance of the factor loads by trying to make each item strongly related to only one factor (minimising overlap).

After rotation, Component 1 is partially ‘moved’ into the space of Component 2. Component 2 better defines an independent part of the latent structure. Component 3 is conditionally ‘separated’ from the other two and represents a very different dimension. The rotated structure is interpretable as the factor loads for each component become more explicit. Component 3 is the most distinct and should be analysed more carefully. After varimax rotation, the factors are more suitable for practical interpretations, such as interpreting scales or dimensions in a given study. As a result of the factor analysis, two factor components emerged instead of three. This can be explained by the similarity between the second and third scales of the questionnaire. Essentially, these scales measure almost the same construct but with a different focus—one is oriented towards work with clients, while the other pertains to the work environment as a whole. After rotation, it can be observed that Component 1 partially ‘shifts’ into the space of Component 2, indicating a strong relationship between these two aspects. Meanwhile, Component 2 more clearly defines an independent part of the latent structure, suggesting a distinct yet important aspect within the scale. Component 3 is relatively ‘separated’ from the other two, indicating that it represents an entirely different dimension. Therefore, it should be analysed in greater depth to understand its significance in the context of the study.

The varimax rotation resulted in a factor structure that is more clearly interpretable, as the factor loadings more distinctly define each component. The rotated structure better supports practical interpretation, such as the analysis of scales or dimensions within the specific study.

Table 8 shows the model goodness-of-fit indices used to assess how well a given model fits the data. Each of the indices provides a specific perspective on model fit and is interpreted based on standards and context.

An SRMR (Standardised Root Mean Square Residual) value below 0.080 is considered a good indicator of suitability. A value of 0.041 indicates an excellent model fit. An RMSEA (Root Mean Square Error of Approximation) value below 0.06 is considered a good fit. The Classical and Scaled variants are within the acceptable range, while the Robust value of 0.139 indicates a poor fit. RMSA (90% TI: 0.055–0.074) indicates good suitability.

Table 9 shows a number of goodness-of-fit indices. The confirmatory factor analysis showed a good model fit: χ^2^/df = 2.60. A CFI (0.997) value above 0.950 indicates an excellent model fit. A TLI (0.997) value above 0.950 is a good indication of a good fit. For NNFI (0.997), similar to TLI, a high value (>0.95) indicates a good fit. For NFI (0.994), a high value (>0.90) indicates a good fit, and 0.994 is close to ideal. For RFI (0.994), similar to NFI, this value indicates excellent fit. For IFI (0.997), a value >0.90 is considered very good. PNFI (0.866) is a conservative indicator and a value above 0.500 is considered good. In conclusion, almost all goodness-of-fit indices (CFI, TLI, NFI, RFI, IFI) indicate an excellent fit of the model. The SRMR values are very low, confirming a good fit. The robust RMSEA (0.139) indicates a poor fit. This indicator should be interpreted with caution. Overall, the model shows an excellent fit with some minor discrepancies (mainly in the robust RMSEA). The KMO (measure of sampling adequacy) was 0.942. All standardized factor loadings exceeded 0.60.

Table 10 details the analysis of the measurement model that explores the three latent dimensions: personal burnout (PRB), work-related burnout (WRB), and client-related burnout (CRB).

PRB, WRB, and CRB are the three latent dimensions that the model attempts to measure based on observed items. Each latent variable is associated with a number of observable variables that reflect the scores on that scale. The estimate indicates the load factor between the latent variables and the observed items. The standard error (SE) helps to assess the precision of the estimate—lower SE values indicate greater precision. The standard error (SE) indicates the range within which the loading coefficient is likely to fall with 95% confidence, while the standardized loading factor allows for comparison of items regardless of their original scale. The z-value indicates the relative importance of the item to the latent variable. A *p*-value (<0.001 for all items) confirms that the load factors are statistically significant.

All items (1–6) are strongly correlated with PRB (β between 0.763 and 0.956), indicating a strong expression of the latent dimension. The loads are statistically significant (*p* < 0.001). Item 10 (−0.660) differs with a negative load coefficient, indicating an inverse association with WRB. For the remaining items (PB7–PB13) the load coefficients are positive and statistically significant (β between 0.796 and 0.904). All items in the CRB (14–19) show positive and statistically significant load coefficients (β between 0.621 and 0.925).

**Scheme 1:**
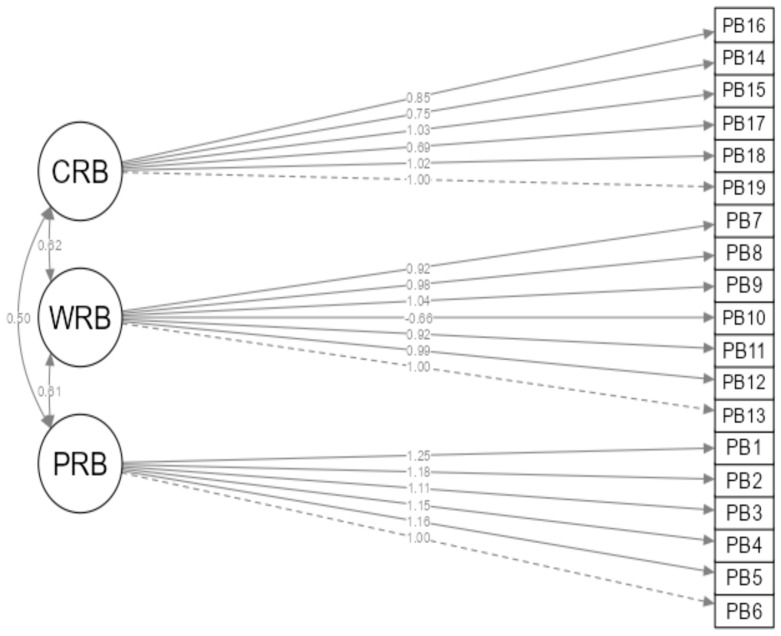
Path diagram.

The correlation between CRB and WRB is high (0.92), indicating a possible overlap in the measurement of these dimensions. PRB correlates with the other two dimensions (WRB = 0.81 and CRB = 0.50). The high correlation between CRB and WRB suggests that these dimensions may be conceptually similar and should be considered analytically and theoretically distinct. The data in Table 10 are also shown in the path diagram (see the Scheme 1).

The numbers on the arrows from the latent variables to the items indicate the strength of the association between them (loading factors). Higher coefficients indicate a stronger relationship. There are correlations between the latent variables (CRB, WRB, PRB). For example, the correlation coefficient between CRB and WRB is 0.92, indicating a strong association.

## 4. Discussion

In this study, the Copenhagen Burnout Inventory was adapted to the Latvian healthcare context in order to provide a reliable and effective tool for assessing burnout. The results indicate that the adapted tool is reliable and appropriate, but also highlight a number of issues that require further evaluation.

In Latvia, previous studies [19,20,21] found that almost 50% of Latvian nurses [21] experienced high levels of emotional burnout, while one-third experienced depersonalisation. These results are comparable to those of our study, where the mean burnout scores in the personal and work burnout dimensions showed moderate to high levels of burnout, and it was nurses who had a higher proportion of respondents—81.3%. The high level of burnout among nurses in Latvia reflects the structural challenges of the sector, including staff shortages [14], high workloads [16], and limited resources [17].

The use of the CBI in different countries [50,51,52,53] shows similar burnout trends, especially in the healthcare sector. The results show that the mean personal burnout score for study participants was 58.9 (SD = 17.3), which is significantly higher than many other populations studied. In a study in Japan [50], respondents working in human care had a mean personal burnout score of 50. In a study of nurses in Greece [51], the mean personal burnout score was 50, which is closer to the Latvian results but still slightly lower. In Maltese nurses [51], the mean score was 54, which is closer to the Latvian results but still slightly lower. In Portugal [53], the average score for nurses was 53. These comparisons show that Latvian healthcare professionals experience significantly higher levels of burnout than their counterparts in other countries. This difference may be related to differences in culture, work organisation [54], and availability of resources [17].

The study found that the mean level of work-related burnout among Latvian healthcare professionals was 56.0 (SD = 15.8). In contrast, in a study of Japanese nurses [50], the mean work burnout score was 43, which is significantly lower than in Latvia. In a study of medical residents in India [55], the mean job burnout score was 54, which is closer to the respondents in our study. In Greece, the job burnout score was 53, while in Malta, it was 51, which is close to but still lower than in Latvia. These results suggest that workload [16] and related stress are higher in Latvia than in many other countries.

The results of the study on the dimension of client-induced burnout had a mean score of 48.9 (SD = 14.3) for the respondents. In contrast, in the Maltese study [51], the client burnout score was 43. In Portugal, the mean client burnout score of the workers was 38. Japanese nurses [50] had a score of 33, which was lower than the Maltese and Portuguese respondents. Latvian respondents scored over 40, indicating an even greater impact of clients on burnout than in many other countries.

Latvian healthcare workers often work long hours [16] to compensate for low salaries, which in turn increases the risk of burnout [56]. Data from our study confirm that on average 42% of respondents experience high workload, which correlates with burnout outcomes. Other countries, such as Greece [51] and Japan [50], have better psychological support systems for healthcare workers than Latvia. It is known that burnout is normalised and that staff do not always seek help, which can be interpreted as low awareness of the consequences of burnout [1,2]. And, of course, there is the cultural factor. Latvia is characterised by a high work ethic, where staff often sacrifice their own well-being for the sake of patient care, leading to burnout in the long term [19]. This situation calls for the systematic implementation of burnout prevention measures, including better work organisation, emotional support, and accessible education on stress management [8,57].

Comparing our results with studies from the USA [33] and Australia [58], the CBI showed high reliability, similar to our study, where the overall Cronbach’s alpha was 0.938. A study in Italy [59] validated the CBI among physicians and found a Cronbach’s alpha ranging from 0.90 to 0.96, indicating high internal consistency, similar to our results in Latvia (0.92–0.94). However, in the Greek study [39], the Cronbach’s alpha was 0.884, lower than in our study but still with high reliability.

Items with low correlations or negative loads (e.g., item 10) have also been identified as potentially problematic in other studies [29,33,39,58], confirming the need to revise these items. The meaning of this item is reversed, which may lead to difficulties in respondent comprehension [10]. Item 10 should be given particular attention in future studies.

Factor analyses in studies in Greece [39] and Spain [29] confirmed the three-factor structure of the CBI but suggested the need to test the relevance of the factors in specific contexts. In our study, factor analysis revealed two defined factors, and when the three factors were analysed, the client-related burnout (CRB) dimension showed a high correlation with the work-related burnout (WRB) dimension, which may indicate an overlap between these dimensions, and these dimensions should be further analysed separately.

Factor analysis showed that the CFI in our study was 0.997, which was higher than in the Greek study [39] with a CFI of 0.938, the Spanish study [29] with a CFI of 0.95, and the Italian study [59] with a CFI of 0.94. The TLI of 0.997 in this study indicated a good fit and was higher than in Greece [39] with 0.923 and in Italy [59] with 0.93. In Latvia, the RMSEA value was 0.064, while in Greece [39], the RMSEA value was higher with 0.074, and the highest value was found in the Italian study [59] with 0.09. The lowest value was 0.013 in Spain [29]. Using SRMR as an index, our study has an index of 0.048, which is similar to the Spanish result of 0.047. Other indices such as NNFI, TLI, NFI, RFI, and IFI showed good or very good fit in our and other countries’ studies [29,39,59].

Although the CBI authors [30] do not recommend the use of fixed cut-off points to measure burnout, in the Latvian context, it could be a useful tool to identify high-risk groups. Empirically based cut-off points should be developed based on data from previous studies and clinical observations. Comparisons with clinical data should be made to determine whether specific CBI scores correlate with increased severity of burnout symptoms and need for intervention. Cut-off points should be tailored to different professions, as burnout thresholds may differ, for example, between healthcare workers and education professionals [2]. And it is important to conduct a validation study that tests different levels of burnout in relation to work productivity, psychological well-being, and health outcomes.

Based on the results of the study, a number of improvements and directions for future research are needed to more accurately assess burnout factors and their impact in different healthcare and other professional settings. To provide more accurate and consistent answers, it is necessary to follow the evolution of burnout over time by analysing how different factors (workload, organisational change, individual psychological resilience) influence changes in burnout levels [2,60]. For example, investigate seasonal variations in burnout, as workload in the healthcare sector can vary according to the time of year and epidemic waves [61]. Analyse individual recovery strategies by assessing which work and personal resources help workers to reduce burnout in the long term. Undertake intervention effectiveness studies to assess the long-term effects of different strategies to reduce burnout (e.g., mindfulness, workload reduction, flexible working) [35].

The study sample consisted mainly of healthcare workers, especially nurses, which limits the generalisability of the results to other professions or sectors. The majority of respondents were women (97.8%), which is typical for the healthcare sector but limits the ability to analyse gender differences in burnout. The majority of participants were from the Riga region (57.15%), which may limit the generalisability of the results to other parts of Latvia, where working conditions and available resources may be different.

The study was conducted using a cross-sectional design, which only allows for analysis of a specific time period and does not allow for causal relationships between burnout factors. Although the psychometric indicators showed high reliability, further validation of the tool is needed through additional studies in different sectors and settings. Although face validity was tested, only five independent assessors participated, which may be an insufficient number for a full assessment.

Although the CBI provides a standardised approach, language and cultural specificities may affect the comparability of results with data from other countries. The authors of the CBI [30] do not recommend the use of cut-off points to define levels of burnout, which can make practical application and data interpretation difficult.

Although the study provides important insights into the levels and causes of burnout in Latvia, these limitations point to the need for further research to fully understand the dynamics of burnout. Future studies should expand the sample range, conduct longitudinal studies, and further improve the validity of the tool, taking into account specific cultural and work environment characteristics. This would help to further strengthen the CBI as a reliable and effective tool for assessing burnout in the Latvian context.

It is important to clarify the questions and identify the cut-off points. Longitudinal studies are needed and a more diverse sample is key to developing more effective burnout reduction strategies and improving working conditions in the professional environment. Future research that takes these recommendations into account could significantly improve understanding of the determinants of burnout and contribute to the development of better policies and working practices. Future studies should assess the applicability and psychometric performance of the Latvian version of the CBI in various professional settings such as education, social care, and the corporate sector.

## 5. Conclusions

The adapted Copenhagen Burnout Inventory shows high reliability, validity and strong model fit indicators in the Latvian context. Most items showed good results, but the negative correlation of item 10 indicates the need for further evaluation. These results confirm the suitability of the tool for assessing burnout among healthcare professionals in Latvia. While the current study focused on healthcare workers, broader validation in other occupational groups is recommended to further confirm the scale’s generalizability and utility. The CBI’s simplicity, cost-free access, and multidimensional structure make it a promising instrument for both clinical use and large-scale research. **Recommendations for use**: apply subscale-level analysis rather than overall scores; use the questionnaire as part of broader assessments, not in isolation; ensure respondents understand reverse-worded items; and conduct periodic re-validation when applying in new populations or settings.

In conclusion, the Latvian version of the CBI is a valid and reliable tool for burnout assessment, contributing significantly to occupational health surveillance and the development of targeted interventions in Latvia.

## Figures and Tables

**Table 1:** Content Validity Index results.

Item Nr.	Experts 1. Phase					Item Nr.	Experts 2. Phase				
	Ex.1.	Ex.2.	Ex.3.	SUM	CVI		Ex.1.	Ex.2.	Ex.3.	SUM	CVI
	Personal burnout						Personal burnout				
1.	1	1	0	2	0.67	1.	1	1	1	3	1
2.	1	1	1	3	1	2.	1	1	1	3	1
3.	1	1	1	3	1	3.	1	1	1	3	1
4.	1	1	1	3	1	4.	1	1	1	3	1
5.	1	1	0	2	0.67	5.	0	1	1	2	0.67
6.	1	1	1	3	1	6.	1	1	1	3	1
	Work-related burnout						Work-related burnout				
7.	1	1	1	3	1	7.	0	1	1	3	0.67
8.	1	1	0	2	0.67	8.	1	1	0	2	0.67
9.	1	1	1	3	1	9.	1	1	1	3	1
10.	1	1	1	3	1	10.	1	1	1	3	1
11.	0	1	0	1	0.33	11.	1	1	0	2	0.67
12.	1	0	0	1	0.33	12.	1	1	0	2	0.67
13.	0	1	1	2	0.67	13.	1	1	1	3	1
	Client-related burnout						Client-related burnout				
14.	0	1	1	2	0.67	14.	1	1	0	2	0.67
15.	1	1	1	3	1	15.	1	1	1	3	1
16.	1	1	1	3	1	16.	1	1	1	3	1
17.	1	1	1	3	1	17.	1	1	1	3	1
18.	1	1	0	1	0.33	18.	1	1	1	3	1
19.	1	1	1	3	1	19.	1	1	1	3	1
CVI (items)					0.807	CVI (items)					0.896
CVI (Ex.)	0.84	0.95	0.68		0.823	CVI (Ex.)	0.895	1	0.789		0.895

**Table 2:** Copenhagen Burnout Inventory in Latvian.

Items	Activities	Never/Almost Never (0%) Nekad/Gandrīz Nekad (0%)	Seldom (25%) Reti (25%)	Sometimes (50%) Dažreiz (50%)	Often (75%) Bieži (75%)	Always (100%) Vienmēr (100%)
Personal burnout						
1.	How often do you feel tired? (Cik bieži Jūs jūtaties izmocīts?)					
2.	How often are you physically exhausted? (Cik bieži Jūs esat fiziski izsmelts?)					
3.	How often are you emotionally exhausted? (Cik bieži Jūs esat emocionāli izsmelts?)					
4.	How often do you think: “I can’t take it anymore?” (Cik bieži domājat: “Es to vairs nevaru izturēt?”)					
5.	How often do you feel worn out? (Cik bieži jūtaties noguris?)					
6.	How often do you feel weak and susceptible to illness? (Cik bieži Jūs jūtaties vājš un uzņēmīgs pret slimībām?)					
Work-related burnout						
7.	Do you feel worn out at the end of the working day? (Vai darba dienas beigās Jūs jūtaties noguris?)					
8.	Are you exhausted in the morning at the thought of another day at work? (Vai no rīta Jūs jūtaties izsmelts, domājot par kārtējo darba dienu?)					
9.	Do you feel that every working hour is tiring for you? (Vai jums šķiet, ka katra darba stunda ir nogurdinoša? )					
10.	Do you have enough energy for family and friends during leisure time? (Vai Jums ir pietiekami daudz enerģijas ģimenei un draugiem brīvajā laikā?)					
11.	Is your work emotionally exhausting? (Vai jūsu darbs ir Jūs emocionāli izsmeļ?)					
12.	Does your work frustrate you? (Vai jūsu darbs Jūs aizkaitina?)					
13.	Do you feel burnt out because of your work? (Vai Jūs jūtaties izdedzis/izdegusi?)					
Client-related burnout						
14.	Do you find it hard to work with clients? (Vai darbs ar klientiem sagādā Jums grūtības?)					
15.	Does it drain your energy to work with clients? (Vai darbā ar klientiem Jūs jūtat enerģijas izsīkumu?)					
16.	Do you find it frustrating to work with clients? Vai darbs ar klientiem Jums sagādā vilšanos?					
17.	Do you feel that you give more than you get back when you work with clients? (Vai jūtat, ka, strādājot ar klientiem, dodat vairāk nekā saņemat pretī?)					
18.	Are you tired of working with clients? (Vai Jūs esat noguris no darba ar klientiem?)					
19.	Do you sometimes wonder how long you will be able to continue working with clients? (Vai Jūs dažreiz domājat, cik ilgi vēl spēsiet turpināt strādāt ar klientiem?)					

**Table 3:** Demographic characteristics of the respondents (n = 288).

Question	n = 288
Gender	Female—282 (97.8%) Male—5 (1.75%) Do not wish to specify—1 (0.35%)
Region of workplace	Riga region—165 (57.15%) Kurzeme region—31 (10.85%) Latgale region—24 (8.3%) Vidzeme region—49 (17.05%) Zemgale region—19 (6.65%)
Family status	Married—144 (50%) Single—59 (20.55%) Divorced—41 (14.15%) Widowed—5 (1.75%) Cohabiting—37 (12.85%) Single with child—2 (0.7%)
Education	Vocational secondary education—31 (10.95%) First level professional higher education—67 (23.15%) Second level professional higher education or Bachelor’s degree—149 (51.65%) Master’s degree—39 (13.55%) Doctoral degree—2 (0.7%)
Position	Medical assistant—2 (0.7%) Nurse—234 (81.3%) Physician assistant—21 (7.35%) Physician—7 (2.35%) Medical resident—6 (2.1%) Physiotherapist—3 (1.05%) Midwife—9 (3.05%) Nurse assistant—2 (0.7%) Dental hygienist—1 (0.35%) Radiologist—3 (1.05%)
Department profile	Outpatient department—86 (29.9%) Intensive care unit—30 (10.4%) Anaesthesia department—30 (10.4%) Therapeutic department—40 (13.9%) Surgery—20 (6.9%) Rehabilitation—8 (2.8%) Paediatrics—10 (3.5%) Diagnostic department—8 (2.8%) Admissions, EMS—10 (3.5%) Psychiatry—26 (9.0%) Dentistry—2 (0.7%) Social care—6 (2.1%) Operating theatre—4 (1.4%) Home care—2 (0.7%) Mental health care—2 (0.7%) Administration—4 (1.4%)
Total workload	Up to 0.25 workload (10 h per week)—2 (0.7%) 0.25–0.5 workload (10–20 h per week)—5 (1.75%) 0.5–0.75 workload (20–30 h per week)—59 (20.55%) 0.75–1.0 workload (30–40 h per week)—140 (48.5%) 1.0–1.25 h (40–50 h per week)—45 (15.65%) 1.25–1.5 h (50–60 h per week)—37 (12.85%)
Working hours	Daily work—119 (41.35%) Shift work—89 (30.95%) Both daily and shift work—80 (27.7%)
Work environment	Outpatient care—89 (30.95%) Inpatient care—171 (59.35%) Home care—3 (1.05%) Both outpatient and inpatient care—15 (5.25%) EMS—6 (2.1%) Management—1 (0.35%) School nurse—3 (1.05%)
Workload in the last month	Very low—6 (2.1%) Low—12 (4.2%) Moderate—89 (30.9%) High—121 (42%) Very high—60 (20.8%)

**Table 4:** Table of statistical indicators for burnout dimensions.

Items	M	SD	Corrected Item-Total Correlation	Item-Rest Correlation	Squared Multiple Correlation	Cronbach’s Alpha if Item Deleted	Scale’s Mean	Cronbach’s Alpha Based on Dimensions
	Personal-related burnout							
**1**	60.24	22.940	0.850	0.803	0.770	0.895	58.9	0.920
**2**	59.72	23.362	0.787	0.738	0.693	0.903		
**3**	63.89	22.042	0.765	0.725	0.636	0.906		
**4**	53.13	24.494	0.775	0.741	0.612	0.905		
**5**	67.19	22.568	0.806	0.755	0.664	0.901		
**6**	49.13	26.630	0.670	0.660	0.481	0.922		
	Work-related burnout							
**7**	73.09	22.298	0.628	0.688	0.522	0.748	56.0	0.791
**8**	54.34	28.206	0.711	0.758	0.633	0.724		
**9**	46.18	25.784	0.785	0.809	0.700	0.712		
**10**	48.09	25.507	−0.489	−0.504	0.271	0.914		
**11**	64.06	25.682	0.739	0.704	0.599	0.722		
**12**	48.78	27.738	0.769	0.781	0.655	0.712		
**13**	57.47	29.080	0.757	0.776	0.640	0.712		
	Client-related burnout							
**14**	41.84	23.019	0.629	0.568	0.476	0.876	48.9	0.884
**15**	48.96	24.136	0.778	0.765	0.672	0.853		
**16**	39.76	22.168	0.700	0.616	0.530	0.866		
**17**	60.42	28.539	0.525	0.501	0.366	0.898		
**18**	50.52	24.951	0.815	0.757	0.722	0.846		
**19**	51.39	27.965	0.789	0.737	0.660	0.850		

**Table 5:** Exploratory factor analysis (EFA).

Items	Factor				
	1	2	3	h^2^	Uniqueness
	Personal-related burnout				
**1**	0.785	0.248	0.317	0.778	0.182
**2**	0.789	0.153	0.295	0.733	0.261
**3**	0.705	0.207	0.340	0.656	0.371
**4**	0.813	0.230	0.084	0.720	0.331
**5**	0.709	0.254	0.340	0.683	0.273
**6**	0.702	0.302	−0.002	0.583	0.516
	Work-related burnout				
**7**	0.641	0.204	0.383	0.599	0.458
**8**	0.725	0.363	0.129	0.675	0.342
**9**	0.738	0.387	0.193	0.732	0.261
**10**	−0.520	0.103	−0.575	0.612	0.651
**11**	0.636	0.361	0.158	0.559	0.404
**12**	0.657	0.541	0.015	0.725	0.223
**13**	0.736	0.341	0.185	0.692	0.328
	Client-related burnout				
**14**	0.246	0.795	−0.029	0.694	0.536
**15**	0.419	0.704	0.281	0.750	0.267
**16**	0.205	0.787	0.166	0.689	0.428
**17**	0.110	0.400	0.698	0.658	0.267
**18**	0.346	0.631	0.537	0.806	0.682
**19**	0.339	0.691	0.402	0.753	0.280
**Eigenvalue after rotation**	7.047	3.991	2.059		
**% of variance**	37,088	21,007	10,639		

**Table 6:** Exploratory factor analysis based on Eigenvalue (EFA).

Items	Factor			
	1	2	h^2^	Uniqueness
**Personal-related burnout**				
**1**	0.815	0.336	0.777	0.212
**2**	0.821	0.239	0.732	0.274
**3**	0.747	0.300	0.648	0.374
**4**	0.785	0.263	0.686	0.345
**5**	0.747	0.346	0.677	0.336
**6**	0.650	0.309	0.519	0.519
**Work-related burnout**				
**7**	0.696	0.307	0.578	0.463
**8**	0.701	0.401	0.652	0.374
**9**	0.727	0.440	0.722	0.312
**10**	−0.654	−0.052	0.431	0.677
**11**	0.621	0.404	0.549	0.492
**12**	0.591	0.544	0.645	0.383
**13**	0.727	0.393	0.683	0.341
**Client-related burnout**				
**14**	0.162	0.770	0.620	0.547
**15**	0.414	0.761	0.750	0.274
**16**	0.171	0.808	0.683	0.459
**17**	0.244	0.560	0.373	0.684
**18**	0.413	0.750	0.733	0.266
**19**	0.367	0.775	0.736	0.284
**Eigenvalue after rotation**	7.281	4.911		
**% of variance**	38,321	25,850		

**Table 7:** Component transformation matrix.

Component	1	2	3
**1**	0.781	0.521	0.344
**2**	−0.567	0.822	0.042
**3**	0.261	0.228	*−0.938*

**Table 8:** Scale goodness-of-fit indices.

		95% Confidence Intervals			
**Type**	**SRMR**	**RMSEA**	**Lower**	**Upper**	**RMSEA *p***
**Classical**	0.048	0.064	0.055	0.074	0.008
**Robust**	0.041	0.139	0.129	0.150	<0.001
**Scaled**	0.041	0.090	0.081	0.098	<0.001

**Table 9:** Scale goodness-of-fit indices II.

Comparative Fit Index (CFI)	0.997
Tucker–Lewis Index (TLI)	0.997
Bentler–Bonett Non-normed Fit Index (NNFI)	0.997
Relative-Noncentrality Index (RNI)	0.997
Bentler–Bonett Normed Fit Index (NFI)	0.994
Bollen’s Relative Fit Index (RFI)	0.994
Bollen’s Incremental Fit Index (IFI)	0.997
Parsimony Normed Fit Index (PNFI)	0.866

**Table 10:** Estimates, measurement model.

95% Confidence Intervals										
Latent	Observed	Estimate	SE	Lower	Upper	β	z	*p*	CR	AVE
**PRB**	**1**	1.252	0.0451	1.164	1.340	0.956	27.8	<0.001	0.91	0.64
	**2**	1.177	0.0396	1.099	1.254	0.898	29.7	<0.001		
	**3**	1.112	0.0406	1.032	1.191	0.848	27.4	<0.001		
	**4**	1.153	0.0403	1.075	1.232	0.880	28.6	<0.001		
	**5**	1.155	0.0371	1.082	1.128	0.882	31.1	<0.001		
	**6**	1.000	0.0000	1.000	1.000	0.763				
**WRB**	**7**	0.919	0.0239	0.872	0.966	0.797	38.5	<0.001	0.88	0.60
	**8**	0.984	0.0250	0.935	1.033	0.853	39.3	<0.001		
	**9**	1.043	0.0200	1.003	1.082	0.904	52.2	<0.001		
	**10**	−0.660	0.0422	−0.743	−0.578	−0.572	−15.6	<0.001		
	**11**	0.918	0.0244	0.870	0.966	0.796	37.6	<0.001		
	**12**	0.985	0.0236	0.939	1.032	0.854	41.7	<0.001		
	**13**	1.000	0.0000	1.000	1.000	0.867				
**CRB**	**14**	0.746	0.0330	0.681	0.811	0.669	22.6	<0.001	0.87	0.58
	**15**	1.031	0.0204	0.991	1.071	0.925	50.5	<0.001		
	**16**	0.846	0.0348	0.777	0.914	0.758	24.3	<0.001		
	**17**	0.693	0.0429	0.609	0.777	0.621	16.2	<0.001		
	**18**	1.023	0.0219	0.980	1.066	0.917	46.6	<0.001		
	**19**	1.000	0.0000	1.000	1.000	0.897				

## Data Availability

The datasets produced and examined in this study can be obtained from the corresponding author upon reasonable request. All data generated or analysed during this study are provided within the published article. The data utilized in this study are confidential.

## Abbreviations

ICD 11	International Classification of Diseases
MBI	Maslach Burnout Inventory
CBI	Copenhagen Burnout Inventory
e.g.,	exempli gratia
CVI	Content Validity Index
IBM SPSS	Statistical Package for the Social Sciences
AMOS	Association for Morbid Obesity Support
M	Mean
SD	Standard Deviation
EFA	Exploratory Factor Analysis
KMO	Kaiser–Meyer–Olkin
CFA	Confirmatory Factor Analysis
CFI	Comparative Fir Index
TLI	Tucker–Lewis Index
RMSEA	Root Mean Square Error of Approximation
PEK	Pētījuma Ētikas komitēja
FVI	Face Validity Index
SRMR	Standardised Root Mean Square Residual
NNFI	Bentler–Bonett Non-normed Fit Index
RNI	Relative-Noncentrality Index
NFI	Bentler–Bonett Normed Fit Index
RFI	Bollen’s Relative Fit Index
IFI	Bollen’s Incremental Fit Index
PNFI	Parsimony Normed Fit Index
SE	Standard Error
CR	Composite Reliability
AVE	Average Variance Extracted
PRB	Personal-related burnout
WRB	Work-related burnout
CRB	Client-related burnout
